# The cervical cancer screening and precancer treatment journey: a qualitative study of experiences among Zambian women living with and without HIV

**DOI:** 10.1093/oncolo/oyaf412

**Published:** 2025-12-13

**Authors:** Misinzo Moono, Anjali Sharma, Julia Bohlius, Mulindi Mwanahamuntu, Katayoun Taghavi, Chanda Mwamba, Ntenje Katota, Kabwe Mwamba, Mwati Chipungu, Esther Hamweemba, Albert Manasyan

**Affiliations:** Reproductive, Maternal, Newborn, and Child Health Department, Centre for Infectious Disease Research in Zambia, Lusaka 10101, Zambia; Social and Behavioural Science Department, Centre for Infectious Disease Research in Zambia, Lusaka 10101, Zambia; Department of Epidemiology and Public Health, Swiss Tropical and Public Health Institute, Allschwil 4123, Switzerland; Department of Public Health, University of Basel, Basel 4001, Switzerland; Women and Newborn Hospital, University Teaching Hospitals, Lusaka 10101, Zambia; Institute of Social and Preventive Medicine, University of Bern, Bern CH-3012, Switzerland; International Agency for Research on Cancer, World Health Organization, Lyon 69366, France; Social and Behavioural Science Department, Centre for Infectious Disease Research in Zambia, Lusaka 10101, Zambia; Social and Behavioural Science Department, Centre for Infectious Disease Research in Zambia, Lusaka 10101, Zambia; Social and Behavioural Science Department, Centre for Infectious Disease Research in Zambia, Lusaka 10101, Zambia; Social and Behavioural Science Department, Centre for Infectious Disease Research in Zambia, Lusaka 10101, Zambia; Social and Behavioural Science Department, Centre for Infectious Disease Research in Zambia, Lusaka 10101, Zambia; Reproductive, Maternal, Newborn, and Child Health Department, Centre for Infectious Disease Research in Zambia, Lusaka 10101, Zambia; Division of Neonatology, School of Medicine, University of Alabama at Birmingham, Birmingham, Alabama 35294-0019, United States

**Keywords:** cervical cancer screening, care-seeking, access, Zambia

## Abstract

**Background:**

Zambia has the third highest cervical cancer incidence rate globally, and it remains the leading cause of cancer-related death among women. We explored the experiences of Zambian women who accessed cervical cancer screening and precancer treatment services to understand factors influencing care-seeking and access across urban, peri-urban, and rural settings.

**Methods:**

In 2020, we conducted eight focus group discussions with women living with and without Human Immunodeficiency Virus (HIV) who underwent cervical cancer screening between 2016 and 2020. To explore their care journey, participants were grouped by screening outcome—those with cervical precancerous lesions and those without. We also conducted 18 in-depth interviews with healthcare workers providing antiretroviral treatment, cervical cancer screening, or precancer treatment at government health facilities. Transcripts were coded and analyzed using thematic analysis.

**Results:**

Care-seeking unfolded across five stages: recognition of need to screen, hesitation and consultation, screening, result interpretation, and treatment. Women were generally knowledgeable about cervical cancer and sought screening promptly, though some delayed due to fear. Social networks and interactions with healthcare workers facilitated screening, while logistical and financial barriers, along with delays in histopathology services particularly in rural areas, hindered access to timely diagnosis and treatment.

**Conclusion:**

Improving cervical cancer screening and precancer treatment in Zambia requires addressing systemic inefficiencies by strengthening laboratory capacity, decentralizing diagnostics, and training healthcare workers to provide respectful, consistent counselling. Expanding community engagement to counter misinformation, leveraging social networks, and providing financial protection are also critical to ensuring timely, equitable, and reassuring care.

Implications for Practice This article outlines a cervical cancer screening and precancer treatment journey, describing women’s decisions, behaviors, and interactions from recognizing the need to screen to receiving care, as well as barriers and facilitators that affect access to screening and precancer treatment services. Findings can inform community sensitization campaigns that counter misinformation and leverage the influence of healthcare workers and social networks to increase screening uptake. In addition, the identification of systemic inefficiencies and diagnostic delays highlight the need for the Ministry of Health of Zambia and other stakeholders to strengthen laboratory capacity, decentralize diagnostic services, and allocate additional resources to improve access to and delivery of cervical cancer screening and precancer treatment services.

## Introduction

Cervical cancer is the fourth most common cancer and the fourth leading cause of cancer-related death among women, with an estimated 662 000 new cases and 348 000 deaths worldwide.^1^ Low- and middle-income countries (LMICs) are disproportionately affected by the disease, accounting for over 80% of the global cervical cancer burden.^2^ Within Southern Africa, Zambia has the third-highest age-standardized cervical cancer incidence and mortality rates of 71.5 and 49.4 per 100 000 women, respectively.^3^ Cervical cancer remains the leading cause of cancer-related death among women in Zambia.^3^ High morbidity and mortality rates are likely driven by the high burden of HIV, limited awareness and knowledge of cervical cancer, poor access to preventative measures, late stage presentation and diagnosis, and inadequate availability of cancer treatment services.^4–7^

To address the high burden of cervical cancer in Zambia, the Cervical Cancer Prevention Program in Zambia (CCPPZ) was established in 2006.^8^ The “see and treat” program integrated cervical cancer prevention services into existing primary health care facilities and capitalized on infrastructure established by the HIV/AIDS care and treatment program.^9^ Nurses were trained to provide free, personalized cervical precancer screening and treatment services.^8^ At the time of our study (2020), the primary screening method was visual inspection with acetic acid (VIA) aided by digital camera enhancement of the cervix (digital cervicography).^8^ Women found to be VIA positive were either offered same-day treatment with cryotherapy or thermal coagulation (small lesions), loop electrosurgical excision procedure (LEEP) (large lesions), or punch biopsy if the lesion appeared clinically suspicious for invasion.^9^ In 2023, the Zambian Ministry of Health introduced Human Papillomavirus (HPV) testing as the primary screening method, followed by VIA. However, scale-up of HPV testing has been slow due to limited resources and supply chain challenges.^10^

Several barriers hinder uptake and access to cervical cancer services, resulting in delays in diagnosis and treatment.^7^^,^^11^ Conversely, facilitating factors have been found to positively influence uptake and access to screening.^12^ Understanding women’s screening and precancer treatment journey is critical to identifying gaps in access and informing strategies to strengthen the continuum of cervical cancer prevention and care. The objective of the study was to explore how women living with and without HIV accessed cervical cancer screening and precancer treatment services, and to examine the factors that influenced their decisions and ability to access care in urban, peri-urban, and rural settings in Zambia.

## Methods

### Data collection

We conducted an exploratory qualitative study across three districts—Lusaka, Chipata, and Lundazi—respectively, representing urban, peri-urban, and rural settings in Zambia. Between September and November 2020, four female Qualitative Research Assistants (QRAs) with extensive training and experience in qualitative data collection, conducted eight focus group discussions (FGDs) with a purposeful sample of women identified through the CCPPZ screening registers as screened for cervical cancer using VIA. Four groups consisted of women with cervical precancerous lesions (WL) and four separate groups consisted of women without lesions (WWL), allowing us to explore their screening and precancer treatment journeys. Each group included women with and without HIV to ensure diversity of experiences.

Additionally, we conducted in-depth interviews (IDIs) with a purposeful sample of 18 healthcare workers (HCWs) from antiretroviral therapy (ART) and cervical cancer screening clinics to explore their experiences in promoting and providing cervical cancer screening and precancer treatment services.

FGDs and IDIs were conducted in private rooms within selected health facilities across the three districts and were digitally audio recorded. FGDs were conducted per participants’ preferred language (Bemba or Nyanja) and were transcribed directly into English in Microsoft Word, while IDIs conducted in English were transcribed verbatim. FGD and HCW IDI topics are detailed in the Supplementary Online Appendix (S1).

### Data analysis

Thematic analysis was conducted using Excel to code the data first deductively using the questioning frame, and then inductively to capture new codes that emerged from the data.^13^ We then categorized themes and subthemes into chronological stages to describe the journey women take as they navigate cervical cancer screening and precancer treatment. Our analysis and interpretation was guided by Kohler’s breast cancer help-seeking pathway,^13^ a conceptual framework that describes how women’s decisions and actions related to breast cancer detection, diagnosis, and treatment are shaped by personal beliefs, social networks, and interactions with the health system. This framework guided the organization and interpretation of participants’ narratives vis-à-vis cervical cancer screening and precancer treatment.

### Ethical considerations

The study was approved by the University of Zambia Biomedical Research Ethics Committee (Ref No. 387-2019) and the National Health Research Authority. All participants provided written consent prior to participation. To compensate for their time and transport, participants received a cash reimbursement.

## Results

### Social demographic characteristics of FGD participants

We recruited 54 women who had previously received screening for cervical precancer between January 2016 and November 2020: 25 women with lesions (WL) and 29 women without lesions (WWL) (Table 1). Most women were married, aged between 31 and 40 years old, and had some secondary level education.

**Table 1.** Social demographic characteristics of women who participated in focus group discussions.

Characteristics	Lusaka *n* = 24	Chipata *n* = 15	Lundazi *n* = 15
**Age**			
** 21-30**	7	2	2
** 31-40**	12	5	5
** 41-50**	3	7	6
*** *51-60**	2	1	2
**Marital status**			
** Single**	6	2	3
** Married**	16	13	7
** Divorced**	1	0	3
** Widowed**	1	0	2
**Highest education level**			
** None**	3	4	1
** Primary (grade 1-7)**	4	0	1
** Secondary (grade 8-12)**	16	9	12
** Tertiary (college/university)**	1	2	1
**Monthly income (ZMW)**			
** <500**	10	11	12
** 500-1500**	7	3	2
** 1501-2500**	3	0	1
** 2501-4000**	1	1	0
** 4001-5500**	0	0	0
** >5500**	1	0	0
** No response**	2	0	0
**Number of children**			
** 0**	10	3	0
** 1**	3	4	2
** 2**	4	1	3
** 3**	2	3	3
** 4**	1	4	1
** 5**	3	0	2
** 6**	1	0	2
** 7**	0	0	2
**Screening status**			
** Women with lesions**	12	7	6
** Women without lesions**	12	8	9

### Cervical cancer screening and precancer treatment journey

The journey that emerged from the FGDs with WL and WWL, supplemented by IDIs with HCWs, comprised five stages. These stages represent distinct phases of women’s experiences as they navigated the cervical cancer screening and precancer treatment process: recognizing the need to screen, hesitating and consulting social networks, undergoing screening, interpreting results, and accessing treatment. The experiences of women and HCWs revealed a complex set of factors that influenced screening and treatment seeking behavior, shaped access to services, and sometimes contributed to delays between stages (Table 2).

**Table 2.** Factors affecting stages of women’s cervical cancer screening and precancer treatment journey.

	Stage 1: recognition of need to screen	Stage 2: hesitation and consultation	Stage 3: screening	Stage 4: interpretation of results	Stage 5: treatment
**Individual**	Experiencing symptoms, knowledge about risks and symptoms, perceived risk and threat, perceived benefit, health seeking attitude	Fear of procedures and outcomes	Feelings of fear and embarrassment, feelings of gratification, experiential knowledge	Economic hardship	Experiential knowledge, feelings of gratification
**Social**	Experiences of cancer-related deaths among family or peers, experiences of benefits of early detection among peers and family, spousal support (emotional/financial/logistical)	Community perceptions about screening procedures and outcomes (misconceptions), social support, screening related experiences among family or peers	Community perceptions about screening procedures and outcomes (misconceptions)	Spousal support (emotional/financial/logistical)	Experiences of successful treatment among family or peers (if diagnosed early), spousal support (emotional/financial/logistical)
**Health system**	Sensitization and health education, mandatory referral by ART clinic, provider recommendation		Wait time, attitude and care of healthcare workers, screening and care processes, trust in biomedical care	Availability of laboratory services, laboratory inefficiencies (result turnaround time), financial constraints	Diagnostic delays, treatment delays
**Structural**		Distance to health facility, transportation costs	Distance to health facility, transportation costs		

#### Stage 1: recognition of need to screen

In the first stage, women recognized the need to screen for cervical cancer either because they experienced symptoms that they interpreted as indicative of cervical cancer, perceived themselves to be at risk, or had been sensitized on the importance of screening.

##### Experiencing symptoms

Women with and without lesions reported experiencing “stomach ache,” “back pain,” “pain when urinating,” “itchy private parts,” “warts,” “vaginal bleeding,” “white discharge,” “painful period pains … like someone who has just aborted,” “pain during sex,” “prolonged period,” “swelling in the vagina,” and “sores on the private parts.” The discomfort caused by these symptoms, along with the perception that they could indicate cervical cancer, motivated women to seek screening. A woman without lesions in Lusaka explained that: “The main reason that made me … screen … I feel pain in the stomach, I have pain even it’s not the monthly periods.” Symptom-driven health seeking was also observed by HCWs at the cervical cancer clinic, with one saying: “Others would come because they feel some abdominal pains because they know that it’s one of the signs. Others may come because they have prolonged PV [per vaginam] bleeding.”

##### Perceived risk and threat

Some women considered themselves “at risk” of cervical cancer due to HIV-infection expressed by one woman (WWL, Lusaka) as “us who are on ARVs [Antiretrovirals].” One woman felt at risk because “Cancer is prevalent in women” (WWL, Lundazi), while another recognized her increased risk of acquiring HPV because her husband was uncircumcised. Additionally, women perceived cervical cancer to be life threatening and emphasized the urgency and importance of undergoing screening giving the example of relatives or friends who had succumbed to cervical cancer: “I saw my neighbour died, she was bleeding a lot, so I decided to go” (WL, Lundazi). Another woman, aware of the impact of delay, disclosed: “I heard that a lot of women are dying, so that’s what made me to go for screening … if they go for screening late, women die” (WL, Lusaka).

##### Sensitization

Many women were knowledgeable about the risks and the symptoms of cervical cancer and of the importance of screening due to health education given at health facilities, during community outreach, and via television, radio, and social media. One woman explained: “I just decided on my own … I used to hear about the lesson that screening is important” (WL, Lundazi). HCWs attested to the positive impact of social media: “The ‘Teal sisters’; it’s a [Facebook] group consisting of women only. It’s meant for women to give each other support, motivation to go and get screened. It helps in boosting the numbers” (HCW, Lusaka).

Women living with HIV (WLWH) in all three settings were prompted to screen primarily due to health education provided at the ART clinic: “I saw from the lessons that we get at the ART clinic … Cervical cancer usually affects women that are positive, who are weakened and are susceptible to many infections” (WWL, Lundazi). In some cases, WLWH were mandated to get screened when accessing ART, as explained by a HCW at an ART clinic in Chipata: “In the women living with HIV … It’s like it has become mandatory for them to be screened … Because of this linkage … between ART and cervical cancer.” For women who were not accessing ART, other mediums such as radio prompted them to screen: “I was listening on the radio, they were talking about cancer … I decided that I would also have to go and get tested” (WL, Chipata).

#### Stage 2: hesitation and consultation

While all FGD participants had been screened, a few women reported that they delayed seeking care due to fear instigated by rumors in their communities. Women overcame their fears and doubts with the support of their social networks.

##### Fear and doubt

Some women feared screening due to negative rumors circulating in the community about the painful insertion of the metal speculum, screening outcomes, the biopsy procedure, and fatalism associated with a cancer diagnosis. The following quotes illustrate the negative impact of the rumors: “They were saying that … the machine [speculum] is painful … and then they cut a piece of your skin, and a scar will remain” (WL, Lusaka). Another woman explained: “I was told that the metal [speculum] for testing for the cancer is what brings the cancer … I refused at that time, I did not get tested …” (WL, Chipata). HCWs confirmed women’s fears: “A lot of them will tell you that they were very scared … Fear of having the disease and because of what they have heard … That once … you have cancer, it means you are going to die” (HCW, Lusaka).

##### Consultation with social network

Some women shared their hesitations with their spouses, family, neighbors, peers, and healthcare workers who eased their fears and encouraged them to get screened. A woman narrated how her mother helped her overcome her fears: “She told me that it was a lie [that the speculum hurts] and that she did not feel any kind of pain… She said that I had to go to the clinic and go and get tested for cancer” (WWL, Lundazi). Another woman was encouraged by a peer who had received early diagnosis and successful treatment: “She is the first person that encouraged me and made me strong to say that I should go … and get tested for cervical cancer” (WL, Chipata). Many women reported that they were supported by their husbands: “He tells me not to be scared, we will know what to do if I have it…” (WWL, Lusaka).

#### Stage 3: screening

To access screening, women needed to find money for transport and cover the distance to the health facility. Upon arrival, HCWs welcomed and counselled them before conducting a guided screening process, which elicited varying emotions and sensations.

##### Distance and transport

Women in urban areas walked to health facilities or found money to take public transport. Additionally, a few women in Lusaka reported that HCWs “gave me money for transport” (WWL, Lusaka) after screening. In comparison, some women in peri-urban and rural areas expressed concerns about the long distance from their homes to the health facility, saying, “we want a place which is near us because I know the distance is the reason why many of us women have fear …” (WL, Chipata). HCWs in the rural areas echoed women’s challenges: “Others, it’s the distance I can say, others they come from very far areas, they don’t have transport money…” (HCW, Lundazi).

##### Positive interaction with healthcare workers

At the health facility, most women in all settings experienced a positive interaction with HCWs, which put them at ease during the procedure and enhanced their trust in the healthcare system. They felt “welcomed”, “encouraged”, “cared for,” and “well received” as explained by a woman with lesions from Lusaka: “They just welcomed me very well … I asked them if I won’t have a problem with conceiving? … They told me that I will just be ok, we were even chatting while laughing.” Similarly, a woman without lesions from Lundazi appreciated that all clients were given the same unbiased care: “I was received quite well, and everyone is treated well. They don’t look at how clean you are or anything.” HCWs recognized the importance of fostering a welcoming environment for women: “You greet, create that rapport and then someone would be comfortable, and they will feel that the nurse or the doctor has a good heart” (HCW, Lusaka).

##### Care procedures

A few women with lesions appreciated the guided screening process. One woman narrated: “… They ask you … to lie on the bed … They tell you what to do to open the legs. Then they just insert the machine [speculum] … then they capture … [the] image. They will show you where that cervix is infected or it’s just okay…” (WL, Lundazi). HCWs confirmed that women appreciated the ability to view images of their cervix. In contrast, no WWL in Lundazi or Chipata described being told when the speculum was being inserted or where the camera was placed, and only a few in Chipata mentioned “being shown” or “watching by myself on TV.”

##### Mixed emotions and sensations

The screening procedure elicited mixed emotions and sensations. A few women felt “shy” or “bad that they were going to see my private parts” (WWL, Lusaka) when asked to undress for the screening. HCWs confirmed that women felt embarrassed stating: “The most difficult part is to make them undress” (HCW, Chipata). Many women reported initially feeling scared due to rumors that insertion of the “metal” is painful, but after screening, they realized that they had been misinformed: “Other women that got tested earlier were scaring us and saying that it hurts when they are testing you. After I … tested, I realized that there was nothing that was painful” (WWL, Chipata).

#### Stage 4: result interpretation

Result turnaround time and interpretation differed for WWL versus WL. All WWL received results immediately and were scheduled for their next screening, whereas WL underwent additional diagnostic procedures and/or treatment. Due to inefficiencies associated with sending cervical biopsies to the central laboratory in Lusaka, WL in Chipata and Lundazi experienced delays in receiving further care.

##### Turnaround time and diagnosis

Aside from one exception in Lusaka, WWL received their results immediately, either while watching the screen or by being shown the “photo” following screening. Women described their results as “just okay,” “negative,” “just clear,” “clean,” “no problem,” and “no cancer.” They “felt good” and “very happy” knowing their results, with one woman declaring: “I felt good because I was told the way I was, and also the way I should take care of myself” (WWL, Lundazi). After screening, women reported appointments ranging from 1-5 years for their next screening.

Women with lesions felt anxious or scared as they waited for their results. Two women explicitly stated that: “They diagnosed me with cancer” and “I was told that my cancer was in the early stages,” while others were found with “sores,” “a problem/small problem” on the cervix or told that their cervix was “not okay” or “had not yet developed to becoming cancer.” As a result, they were told that they had to undergo cervical biopsy: “A piece had to be cut so that they test it for cancer,” or treatment—“They had to burn it.” One woman received a paper copy of the results without any explanation of their implications, while several other were still waiting for their results.

Due to the lack of histopathology services at the government labs, health facilities in Eastern Province (Chipata and Lundazi) sent cervical biopsy samples to the central laboratory in Lusaka. Many women in Lundazi and Chipata stated that their histopathology results were either delayed or lost: “It is not known when the results will come, other results are lost” (WL, Lundazi). HCWs in Chipata concurred with these reports, with one saying: “Sometimes we just take those samples, and they go for good … We usually don’t get results.”

##### Financial barriers

Due to the inefficiencies at the government laboratory, HCWs often advised women to pay out of pocket for histopathology testing of their cervical biopsy samples at a private laboratory: “Most of them we tell them if they can afford, they can pay because it’s better to know than not to know.” However, many women reported that they could not find the funds: “I was told … that a piece had to be cut so that they test it for cancer, [but] I don’t have money” (WL, Chipata). Women reported that if they did not find the funds, their samples were not tested: “When they get the sample, the sample stays that side unless you find the money” (WL, Lundazi).

#### Stage 5: treatment

Women found with precancerous lesions that did not require additional diagnostic testing were either treated the same day or referred to see a doctor on another day. Conversely, WL who required additional testing, but experienced delays or loss of their biopsy results were unable to proceed with treatment.

##### Treatment procedures

A few WL in Lusaka believed that cancer is treatable if diagnosed early: “They give pills to take if it’s in its early stages” (WL, Lusaka). Women who were eligible for same-day treatment described procedures such as “spraying,” “laser beams,” “burning,” and “pills.” One woman explained: “I was told that there was something on my cervix that we are going to need to burn off, but it was done the same day” (WL, Chipata). Aside for one woman in Lusaka who felt “cold and pain inside” during treatment, no other women reported feeling any pain, disproving contrary rumors.

After treatment, women felt “good,” “restored,” “cured,” and believed that the procedure was successful in relieving symptoms: “I felt good, the pain that I had in the stomach was no more and my body was even restored” (WL, Lusaka). Except for one woman in Lusaka, no other women faced challenges from their husbands regarding abstaining from sexual intercourse following treatment.

##### Delayed treatment

A few women in Lundazi were unable to proceed with treatment because they had not received their histopathology results. One woman shared: “I’m still waiting for the doctor to remove the uterus because they said that they need the results first …” (WL, Lundazi), and another woman expressed: “Others, they die before those results come in” (WL, Lundazi).

## Discussion

The cervical cancer cascade in Zambia is a nonlinear journey shaped by individual, social, health system, and structural factors. The journey unfolds across five interconnected stages. At each stage, multiple breakdowns can occur, including misinformation and fear, limited transport and facility access, weak laboratory infrastructure leading to delays in diagnostic results, and financial constraints. These challenges particularly affect women living in rural or peri-urban areas. Strengthening each component of this cascade is essential to improving the timely detection and treatment of cervical cancer thereby reducing preventable morbidity and mortality among Zambian women.

Our study found that many women sought cervical cancer screening because they perceived themselves to be at risk, associated their symptoms with those indicative of cervical cancer, recognized the importance of screening, and understood the consequences of delayed care seeking. While screening programs emphasize prevention, many women still linked the decision to screen with the presence of symptoms, echoing evidence that symptom-driven care-seeking remains common in low- and middle-income countries.^14–18^ Women living with HIV were particularly proactive in screening, partly due to systematic integration of screening within ART clinics,^9^ demonstrating the value of leveraging existing health platforms for preventive services. Facilitators to screening such as knowledge and awareness of cervical cancer, high-risk perception due to HIV infection, and the presence of symptoms have been reported in studies in Zambia^19^ and other African countries.^18^^,^^20^

While many women sought screening promptly, others delayed due to fear driven by community misconceptions about the screening process and its outcomes, a finding consistent with other studies from Zambia and other LMICs.^19^^,^^21–23^ These delays reflect the influence of community-level rumors and insufficient or inconsistent counselling at health facilities, leading to women’s embodied experiences of pain, fear, and reassurance. To improve screening uptake, strategies should include awareness campaigns that emphasize the preventive nature of screening to shift women away from symptom-driven care-seeking. Leveraging ART clinics and community support groups to promote screening and dispel misconceptions may be particularly effective.

Although fear initially deterred some women, encouragement from social networks, especially peers and family, ultimately facilitated care-seeking. This finding aligns with studies from Zambia and other African countries which highlight the influential role of partners and family in women’s health decisions.^19^^,^^24–27^ Targeted sensitization campaigns should include messaging for men, particularly husbands, to enhance support for women’s screening.^24–29^

Geographic and infrastructural disparities directly undermine women’s survival prospects. Women in peri-urban and rural areas cited long distances to health facilities, and lack of money for transport as barriers to accessing care. These findings align with studies from Malawi, Peru, and Uganda that identified similar barriers to accessibility of cervical cancer services.^30^^,^^31^ Mobile community outreach could improve accessibility by lowering transportation needs and costs.

Our findings suggest that women generally had positive cervical cancer screening experiences, with HCWs playing a central role in guiding them through the process. HCWs were aware of how their attitudes and interactions influenced women’s experiences, and therefore, made deliberate efforts during counselling and screening to ensure respectful and supportive care. These positive interactions and a well-guided screening process alleviated common concerns such as embarrassment related to “nakedness,” anticipated pain, and misconceptions that the speculum causes cancer, issues also identified in other studies from African countries.^16^^,^^24^^,^^32^ HCWs should actively monitor women’s screening experiences to identify and address any areas of concerns and encourage them to return for follow-up screening visits.

Delays in diagnosis and treatment were reported in rural areas primarily due to laboratory inefficiencies and financial constraints. Although cervical cancer services are free in Zambia, HCWs often advised women with lesions to seek histopathology testing at a private laboratory. However, many women could not afford the high costs at the private lab, resulting in untested samples. As found in Rwanda and Uganda, diagnostic delays occurred due to long wait times for laboratory results, loss of biopsy samples or results, inadequate health infrastructure, and lack of pathologists.^32–34^ It is crucial that the Zambian health system provides efficient diagnostic services, including decentralized laboratories, reliable specimen transport, and subsidized private testing.

A key strength of this study is its inclusion of women both with and without precancerous lesions across urban, peri-urban, and rural settings, as well as triangulation of healthcare worker perspectives, providing a comprehensive view of the screening and precancer treatment journey. However, as a qualitative study, the findings are not necessarily generalizable to larger populations of women or to other districts within Zambia. Additionally, we did not distinguish between WLHV and other women in the FGDs.

## Conclusion

By focusing on the lived experience of women and healthcare workers, this study underscores that systemic inefficiencies are not neutral technical challenges but drivers of suffering, inequity, and preventable mortality. Addressing these requires not only strengthening laboratory capacity and decentralizing diagnostic services but also rethinking how care is organized, communicated, and supported. Investment in community engagement to counter misinformation, in healthcare worker training to sustain respectful and consistent counselling, and in financial protection mechanisms to prevent catastrophic costs are all critical to ensuring that women experience a care journey that is timely, reassuring, and equitable.

## Supplementary Material

oyaf412_Supplementary_Data

## Data Availability

The data underlying this article will be shared on reasonable request to the corresponding author.
