# Supplementary material for: The cervical cancer screening and precancer treatment journey: a qualitative study of experiences among Zambian women living with and without HIV
Source: Oncologist. 2025 Dec 13;31(1):oyaf412. doi: 10.1093/oncolo/oyaf412 (PMC12782829; doi:10.1093/oncolo/oyaf412)
Supplement: oyaf412_Supplementary_Data [file oyaf412_supplementary_data.zip › IDI Guide for HCP English v1.0 20 June 2019.pdf]

IDI GUIDE FOR HCP

To begin with could you tell me about your work with cancer services at this facility, how long have you been in this role?

General information and observations made in Cervical Cancer screening services

- In your opinion, what is the average number of women who come for Cervical Cancer screening per week?
- In your opinion, what is the age group of most women who come for CC screening?
- What brings the women to clinic? (routine screening, self-referrals, provider-initiated referrals, routine follow-up)
- Who accompanies the women to the clinic?
- Have you seen any changes in trends in the number and type of women coming for screening?
    - What are these changes?
    - What do you think brought these changes (or not)?

Information shared during referral

- What sort of information do you share with the women before you refer them for cancer screening?
- How do you know if women understand what you explain?
    - What do you do if one does not understand what you are trying to explain about the need to/procedures for screening?
- How much do the women know about screening procedures by the time you see them?
- What do they know?
- What feelings/thoughts do they share with you?
- What is the most difficult part of the referral process for the women to cancer screening?

Information during cancer screening process

- Where do you think women get information on Cervical Cancer screening from?
- When women are waiting for Cervical Cancer screening, what do they talk about to each other?
- What sort of information is provided in the waiting room? (pamphlet, Audio-Visual)
- What are some of the questions the women ask prior to the actual screening?
- Could you share what you tell the women before the actual screening procedure?
- How do you know if women understand what you explain?
- What feelings/thoughts do they share with you?
- What challenges do you face in sharing information with women about cervical screening?

Perceptions on knowledge and health seeking behavior of women

- How do women explain their complaints related to their genitalia?
- What signs and symptoms do they normally report?
- What procedures do they try at home or community before coming to the facility?
- What delays women to seek care for these symptoms?
- What delays women from accessing Cervical Cancer screening?
- What are the main reasons women resort to Cervical Cancer screening?
- Why do you think some women with complaints about their cervix never resort to screening? (perceptions on screening, cancer and cancer treatment)
- What challenges do you think women face in accessing information about cervical cancer screening?

Procedures in the Cervical Cancer screening services

- Can you describe the procedure before the screening process? (How they are welcomed, what information is given)
- Could you describe the screening procedure?
- How long do results take? How are they shared?
- How is "bad" news (found with lesions) told to women?
- How do women react to "bad" news?
- What is the procedure for women found with cervical cancer?
    - How are they followed-up?
    - What support is available
- What challenges do you think women face in accessing CC screening services?

Recommendations on improving demand for Cervical Cancer screening

- Which kind of organizations or individuals should be assigned for informing women on the need for Cervical Cancer screening?
- What could be done to increase the women's demand for Cervical Cancer screening?
- What kind of role should Cervical Cancer staff play?
- What kind of role should ART staff play?
- What would be your recommendations for improving the information on Cervical Cancer screening?
- How could ART clinics contribute to a better understanding among women of the need for Cervical Cancer screening?
- How could Cervical Cancer screening clinics contribute to a better understanding among women of the need for Cervical Cancer screening?
- Other than women, who else needs information on Cervical Cancer? (men)
    - What kind of messages would you suggest?

- From whom? and how?
