# Supplementary material for: The cervical cancer screening and precancer treatment journey: a qualitative study of experiences among Zambian women living with and without HIV
Source: Oncologist. 2025 Dec 13;31(1):oyaf412. doi: 10.1093/oncolo/oyaf412 (PMC12782829; doi:10.1093/oncolo/oyaf412)
Supplement: oyaf412_Supplementary_Data [file oyaf412_supplementary_data.zip › FGD Guide for Screened Women English_ v1.0 20 June 2019.pdf]

SAMPLE FGD GUIDE 2 FOR WOMEN SCREENED FOR CANCER IN CLINIC SETTINGS

We will be talking mainly about your experiences and thoughts about Cervical Cancer screening and Cervical Cancer treatment. Firstly, I'd like to hear about *things* for which women might go to see a doctor or nurse. Some women may go to a doctor or nurse fairly often; while others may not.

- How often do women go to see a doctor or nurse for themselves – not children, parents or others?
- For what sorts of things do women seek a doctor or nurse? (probe: symptoms, prevention, both, other)
- What are the different types of tests women can receive at the facility that you go to?
- How do women feel about these tests? (necessary, timely, results, comfort)

Everyone here has had at least one cervical screening. Let's talk about your experiences.

- What is the doctor or nurse looking for when she or he examines your cervix?
- What had you heard about Cervical Cancer screening before your first screening?
- What did you know about the experience of a woman who has gone through Cervical Cancer screening before you were screened?
- Did you consult anyone about the decision to be screened?
    - Who did you consult?
    - Why did you consult them?

Now I would like you to think back to Your LAST Cervical Cancer screening –

- What was the main reason you decided to go? (probe: reminder, social support, social pressure, motivated)
- How was your experience in getting an appointment?
- What arrangements did you have to make at work and home to make the appointment?
- What arrangements did you have to make to get to the facility? (transport, money, food, company)
- How did the staff treat you?
- How did they handle your questions?
- How long did you wait at the facility for you to be attended to and screened?
- What was your experience during the screening?
    - How did the clinic describe the procedure?
    - How did you feel waiting to be screened? (feelings, thoughts, fear, anxiety)
    - What did if anything, the women in the waiting room discuss?
    - Did you talk to other women in the waiting room? If so, what did you discuss?

    - How did the perceptions of other women make you feel about proceeding with the screening?
    - How did they handle your body?
    - How did you feel waiting for your results? (feelings, thoughts, fear, anxiety)
    - How did you feel after being screened?
- What do you think of the place where you were screened from? What would be your ideal Cervical Cancer screening centre – (one where you would be most comfortable and would recommend to other women)

Suppose your friend had never had Cervical Cancer *screening*, what would you tell her about your experience.

*FOR WOMEN WHO SAY THEY **DID NOT** OBTAIN RESULTS:*
- Do you have any ideas about why you *did not* get the results?
- What did you do to find out if your results were ready?

*FOR WOMEN WHO SAY THEY **DID** RECEIVE RESULTS:*
- How did you hear about the results of your Cervical Cancer *screening*?
- How were you told? (verbally, in writing, time taken) How soon?
- How did you feel when you received your results?
- What did you do about things that were confusing or hard to understand about the results?
- What were you told after receiving your results?
- How did you feel about the information you were given while receiving the results?
- Overall how do you feel about the screening procedure you underwent?

For those (found with lesions) What was your experience after getting the results? (support, information given, challenges)

What was your experience with the treatment?
- What arrangements did you have to make at work and home for the treatment?
- What arrangements did you have to make to get to the facility? (transport, money, food, company)
- How did the staff treat you?
- What support did you receive during treatment (from whom)?
- Did you seek treatment help from anywhere else outside the clinic? (where, why)
- Overall how do you feel about the care you received? (medication availability, communication, care processes)

What advice would you give to the clinics to improve in the treatment of cancer?
What can be done to help women obtain Cervical Cancer treatment?

For those (found without lesions) What was your experience after getting the results? (support, information given, challenges)

- What have you heard about those on treatment?
- What types of cancer treatment have you heard of? (traditional, herbal)

Reasons women may not go back for re-screening.

- Please tell me about why women may not go back to be re-screened. (experience, perceived need, convenience, staff, pain, embarrassment, cost)
- What can be done to help women return for re-screening?

Reasons women may go back for re-screening

- Some women do go back for Cervical Cancer re-screening; could you share with me some reasons why some women may go back to be re-screened? (experience, perceived need, convenience, staff, family support, peer pressure)
- Does Cervical Cancer screening experience get better with time?
